# Enhanced Degradability, Mechanical Properties, and Flame Retardation of Poly(Lactic Acid) Composite with New Zealand Jade (Pounamu) Particles

**DOI:** 10.3390/polym15153270

**Published:** 2023-08-01

**Authors:** Lilian Lin, Quang A. Dang, Heon E. Park

**Affiliations:** 1 Department of Chemical and Process Engineering, University of Canterbury, Christchurch 8041, New Zealand; lilian.lin@pg.canterbury.ac.nz; 2 New Zealand Institute for Minerals to Materials Research, Greymouth 7805, New Zealand

**Keywords:** 3D printing, compression molding, biodegradable plastic, New Zealand jade, pounamu, thermal stability, mechanical strengths, rheology, Cox-Merz rule, Berry-Fox equation

## Abstract

Plastic pollution has become a global concern, demanding urgent attention and concerted efforts to mitigate its environmental impacts. Biodegradable plastics have emerged as a potential solution, offering the prospect of reduced harm through degradation over time. However, the lower mechanical strength and slower degradation process of biodegradable plastics have hindered their widespread adoption. In this study, we investigate the incorporation of New Zealand (NZ) jade (pounamu) particles into poly(lactic acid) (PLA) to enhance the performance of the resulting composite. We aim to improve mechanical strength, flame retardation, and degradability. The material properties and compatibility with 3D printing technology were examined through a series of characterization techniques, including X-ray diffraction, dispersive X-ray fluorescence spectrometry, scanning electron microscopy, energy-dispersive X-ray spectroscopy, thermogravimetric analysis, 3D printing, compression molding, pycnometry, rheometry, tensile tests, three-point bending, and flammability testing. Our findings demonstrate that the addition of NZ jade particles significantly affects the density, thermal stability, and mechanical properties of the composites. Compounding NZ jade shows two different changes in thermal stability. It reduces flammability suggesting potential flame-retardant properties, and it accelerates the thermal degradation process as observed from the thermogravimetric analysis and the inferred decrease in molecular weight through rheometry. Thus, the presence of jade particles can also have the potential to enhance biodegradation, although further research is needed to assess its impact. The mechanical properties differ between compression-molded and 3D-printed samples, with compression-molded composites exhibiting higher strength and stiffness. Increasing jade content in composites further enhances their mechanical performance. Th results of this study contribute to the development of sustainable solutions for plastic pollution, paving the way for innovative applications and a cleaner environment.

## 1. Introduction

There is no need to state the obvious any longer—the detrimental impact of plastic pollution on our environment has reached an apparent level. It is a pressing global issue that requires immediate attention and concerted efforts to mitigate its far-reaching consequences. Even in regions as remote as New Zealand [1], a country geographically distant from many others, the presence of microplastics has been detected, underscoring the pervasiveness of this problem.

Recognizing the urgency to address plastic pollution, researchers and scientists have been exploring various potential solutions. One such avenue of investigation involves the development and utilization of biodegradable plastics [2]. These plastics have gained considerable attention as a promising alternative, offering the prospect of reduced environmental harm through their ability to degrade over time. However, it is important to acknowledge that biodegradable plastics also have their own set of limitations and challenges to overcome. Two noteworthy limitations of biodegradable plastics warrant attention. Firstly, compared to conventional plastics, biodegradable counterparts often exhibit lower mechanical strength, which can impact their practical applications in numerous industries. Secondly, the degradation process of biodegradable plastics can be slower than initially anticipated, raising concerns about their overall effectiveness in mitigating environmental pollution. Consequently, both of these factors contribute to the slow adoption and integration of biodegradable plastics into mainstream usage.

To address these limitations, our study aims to investigate the incorporation of New Zealand jade (pounamu) particles into poly(lactic acid). PLA is one of the most commonly used synthetic bio-based plastics in the industry and research for applications including three-dimensional (3D) printing [3,4,5], fabrics, packaging/bottles, films, automotive, electronics, and tissue engineering [6]. The properties of some PLA grades are similar to various non-degradable plastics. More than 200,000 patents have been published [7]. The number of relevant publications has increased exponentially such as for phase and stereocomplex behavior [8,9,10,11], rheology [12,13,14,15], foaming [16,17,18,19,20], recycling [3,21,22,23], and composites [5,24,25,26]. There are a number of review papers and monographs [6,7,7,27,28,29,30,31,32,33,34,35,36,37,38,39]. To enhance a specific property of PLA, inorganic fillers are often added as minor components, such as nanoparticles [25], talc [40], calcium carbonate [41], mica [42], and various mineral fillers [43]. However, none of these have been reported to simultaneously enhance the multiple properties that we aim to increase. Thus, our objective in compounding PLA with NZ jade particles is to enhance the overall performance of the resulting composite. Specifically, we aim to improve its mechanical strength, enhance flame retardation, (i.e., reduce flammability), and increase degradability. In addition to examining the material properties of the composite, we also explore its compatibility with 3D printing technology and compare this with compression molding. To investigate these aspects, we conducted a series of preparation processes and characterizations, including X-ray diffraction, dispersive X-ray fluorescence spectrometry, scanning electron microscopy, energy-dispersive X-ray spectroscopy, thermogravimetric analysis, 3D printing, compression molding, pycnometry, rheometry, tensile tests, three-point bending, and flammability tests. This comprehensive investigation provides valuable insights into the potential applications of the composite in advanced manufacturing processes. Furthermore, we conducted rheological studies to assess the feasibility and effectiveness of the composite material, specifically in terms of its thermal stability and decreased molecular weight, hoping that the composites have accelerated biodegradability. By undertaking this research, our aim is to contribute to the ongoing efforts in developing sustainable and environmentally friendly solutions to address plastic pollution. Through an improved understanding of the properties and performance of the compounded composite, we can pave the way for innovative applications and contribute to a cleaner and healthier environment for future generations.

## 2. Materials and Methods

### 2.1. Materials

Poly(lactic acid) (PLA) was selected as a based polymer matrix to fabricate composites due to its biodegradability and production of final products with fair precision tolerance [44]. To fabricate the composites, a grade of PLA (Ingeo, Natureworks 2003D, Minneapolis, MN, USA) was selected. This polymer has a melt flow index of 3.2 g/10 min at 190 °C and 2.16 kg, a D-Lactide content of 4.3% [45], and a weight-average molecular weight (*M*_w_) of 230,000 g/mol [46]. As a reinforcing member, New Zealand jade particles (Waewae Pounamu shop, Hokitika, New Zealand) were obtained. Nitrogen (N_2_, BOC Gas & Gear, Christchurch, New Zealand, >99.99% purity, instrument grade) was used to create an oxygen-free environment for the thermogravimetric analysis and rheometry. Helium (He, BOC Gas & Gear, New Zealand, >99.8% purity, instrument grade) was used for the pycnometry.

### 2.2. New Zealand Jade

**Figure 1:**
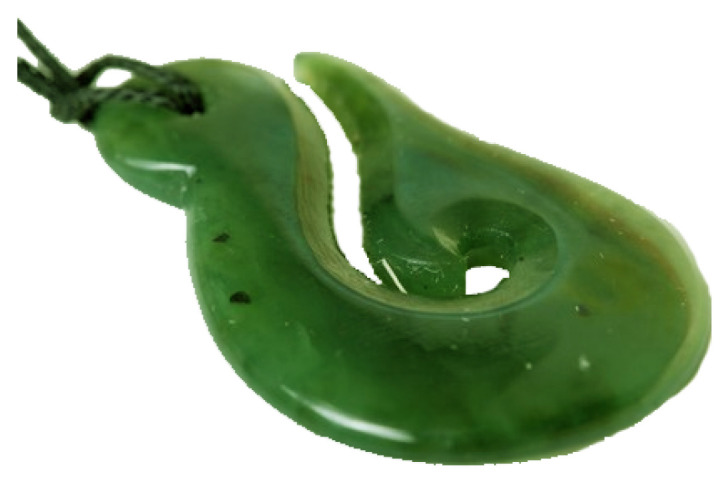
A fishhook (hei matau) made of NZ jade meaning strength, prosperity, peace, good luck, and safe journey over water.

Jade encompasses a wide range of green-white gemstones, with New Zealand (NZ) jade commonly known as “greenstone” among early European settlers and “pounamu” by the indigenous Māori people, symbolizing its cultural significance. Within the realm of pounamu, there are primarily two types: nephrite jade [47], commonly referred to as greenstone, which is highly valued for carving, and translucent bowenite. NZ jade is predominantly sourced from the West Coast region of the South Island in New Zealand. It holds deep cultural importance as a symbol of status and power. Traditionally, NZ jade has been intricately carved and prominently displayed to depict Māori narratives. It has also been fashioned into tools and weapons for historical warfare. Various shapes of carved NZ jade, each carrying its unique meaning, have become one of the most popular souvenirs in New Zealand (Figure 1). Throughout the carving process, a significant amount of fine and coarse NZ jade particles is generated, accounting for approximately 20–30% of the waste produced by carvers, artists, and designers. Recognizing the potential of these NZ jade particles, specifically nephrite jade, as a functional additive in the development of biodegradable composite materials using additive manufacturing techniques, we refer to them interchangeably as “NZ jade”, “jade particles”, or “pounamu” throughout this paper.

## 3. Methods

**Figure 2:**
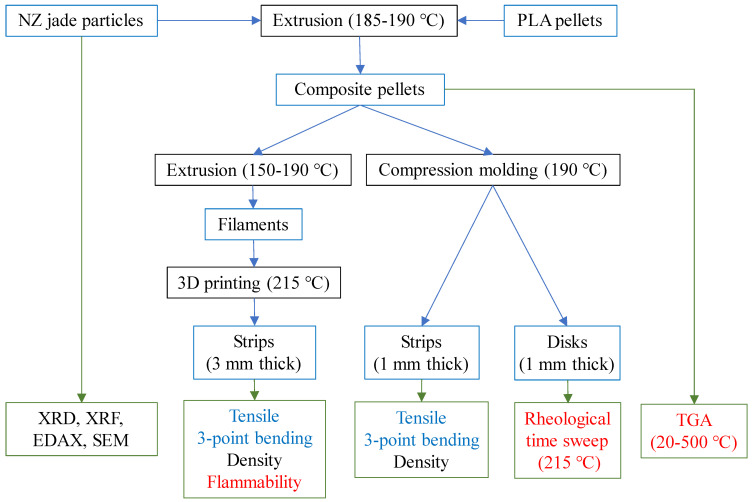
The composite preparation and characterization methods. Characterization methods can be categorized into three groups based on their focus: chemical and density (in black), thermal stability (in red), and mechanical properties (in light blue).

We have employed a variety of methods for sample preparation and characterization, as illustrated in Figure 2 to achieve our aim in this study.

### 3.1. NZ Jade Analysis

#### 3.1.1. Screening NZ Jade Particles

To refine the size distribution of the NZ jade particles, a sieve analysis method was employed. The raw jade particles obtained from the supplier, who ground NZ jade to produce particles, underwent a drying process in a vacuum oven (8100, Contherm Scientific, Lower Hutt, New Zealand) at 105 °C for 24 h. After drying, the particles were loaded onto an electromagnetic sieve shaker (EML 200 Premium, Haver, Oelde, Germany), which consisted of a series of sieves arranged in decreasing mesh diameters (250, 212, 106, 75, and 38 µm) from top to bottom. The sieve shaker was operated with vibrations, causing the jade particles to segregate onto different-sized sieves based on their particle size. For this study, the particles collected from the sieve with a mesh diameter of 38 µm were selected and utilized.

#### 3.1.2. Mineralogy of NZ Jade

X-ray diffraction (XRD) analysis was performed to determine the mineralogy of the dried NZ jade particles and identify the specific type of rock present. The fine jade particles were dried in a vacuum oven at 105 °C to eliminate any moisture. An X’Pert Pro XRD instrument (Phillips, Amsterdam, The Netherlands) equipped with parallel beam optics and Co Kα radiation was employed in the XRD analysis for the rock type analysis. To determine the chemical composition, specifically the oxide contents, a PW2400 sequential wavelength dispersive X-ray fluorescence spectrometer (XRF, Phillips, The Netherlands) was used. The XRF analysis provided information on the contents of ten oxides. The element compositions of NZ jade were analyzed using energy-dispersive X-ray spectroscopy (EDAX) with a JSM-IT300 instrument (JEOL, Akishima, Japan). EDAX analysis offers valuable insights into the elemental composition and distribution within the jade particles, aiding in the characterization of their properties. The average values were obtained by analyzing four different particles using EDAX.

### 3.2. Composite Preparation (Extrusion Compounding)

The raw PLA pellets, provided by the manufacturer, were subjected to a drying process in the vacuum oven at 45 °C overnight to eliminate moisture and volatiles before extrusion compounding. Four PLA/jade composites (Table 1) were prepared by compounding the materials using a twin-screw extruder (Labtech Engineering Co., Ltd., Mueang Samut Prakan, Thailand) equipped with a 2 mm 3-hole die, following the conditions specified in Table 2. First, the polymer pellets and NZ jade particles were manually mixed, and then the mixture was fed into the extruder’s hopper feeder. After successful extrusion of the strands, they were cooled in a water bath and subsequently pelletized to produced cylindrical pellets. The resulting pellets underwent another round of overnight drying in the vacuum oven at 45 °C to ensure the removal of any remaining moisture and volatiles. These composite pellets served as the raw material for filament fabrication for 3D printing or for the compression molding of disks and strips.

### 3.3. Filament Fabrication for 3D Printing

The pellets of the four composites were individually processed using the Labtech twin-screw extruder equipped with a 2 mm 1-hole die, following the conditions specified in Table 3. After the extrusion process was completed, the resulting filaments were cooled in a water bath and then wound onto spools suitable for 3D printing. The targeted filament diameter was 1.75 mm, specifically designed for use in the 3D printer. To ensure optimal printing performance, the spools containing the filaments underwent additional drying in the vacuum oven at 45 °C for at least one day before commencing the 3D printing process.

### 3.4. 3D Printing Strips

For the tensile and three-point bending tests, density measurements, and flammability tests, composite strips were fabricated using an M2 3D printer (Makergear, Ltd., Beachwood, OH, USA) equipped with a 0.75 mm nozzle. The printing parameters included an extrusion temperature of 215 °C and a bed temperature of 65 °C. The composite strips were printed with a layer thickness of 0.2 mm, 100% infill, and an orientation angle of −45°/45°. The dimensions of the strips were 125 mm×12.7 mm×3.2 mm. To ensure consistent material properties, all strip composites were stored in a vacuum oven (OV11, Jeiotech, Daejeon, Republic of Korea) at room temperature for at least one week before conducting the measurements.

### 3.5. Compression Molding Disks and Strips

After drying the pellets of the four composites in the vacuum oven (OV11, Jeiotech, Republic of Korea) at room temperature for over a week, they underwent reprocessing by compression molding to fabricate disks for rheological tests and strips for tensile and three-point bending tests and density measurements. Each composite was individually compression-molded using a hot press (model 3912, Carver, Inc., Wabash, IN, USA) with a 25 mm diameter and 1 mm thickness mold or a 125 mm×125 mm×1 mm mold. Strips were cut to be 125 mm×10 mm×1 mm. The compression molding was performed under a force of 5 tons and a temperature of 190 °C. Following the molding process, all the molded composites were stored in the vacuum oven at room temperature for at least an additional week before conducting the rheological measurements.

### 3.6. Density

The density of the 3D-printed strips and compression-molded strips was measured using a pycnometer (UltraFoam, Quantachrome Instruments, Boynton Beach FL, USA) at a temperature of 20 °C using helium at a pressure of 0.4 bar. The instrument obtained averages over three repetitions, with a standard deviation below 0.02 g/mL.

### 3.7. Thermal Stability

We conducted three different thermal stability tests on the composites. These were to assess the impact of compounding NZ jade with the PLA matrix on the thermal stability of their composites in three different aspects.

#### 3.7.1. Flammability

We conducted flammability tests following the ASTM D635-22 Standard Test Method for Rate of Burning and/or Extent and Time of Burning of Plastics in a Horizontal Position [48]. Prior to testing, the composite strips were stored in the vacuum oven (OV11) at room temperature for over a week. In summary, a 3D-printed strip, with two marks at 25 mm and 100 mm from one end, was horizontally mounted in a fume hood. A gas torch flame was applied to the end of the strip to a depth of 6 mm, and the timing started simultaneously. The flame was withdrawn from the strip after either 30 s or when the flame front reached 25 mm distance from the end, whichever occurred first. In this study, the flame front passed the 25 mm reference mark but did not reach the 100 mm reference mark. The elapsed time (t) in seconds and the burned length ($L_{B}$) in millimeters between the 25 mm reference mark and the point where the flame front stopped were recorded. To ensure accuracy and reliability, each test was repeated three times for each type of composite. The linear burning rate (v) is calculated by:

(1) $$v=\frac{60L_{B}}{t}\;\left(\frac{mm}{min}\right)$$

**Figure 3:**
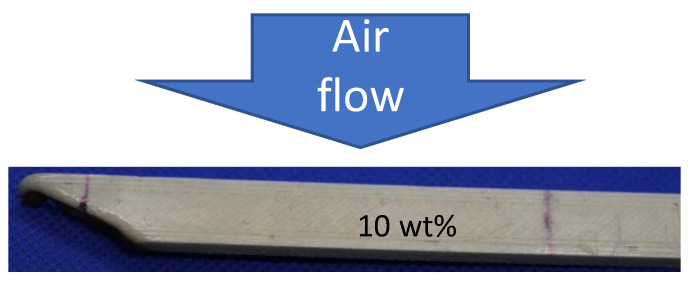
After conducting the flammability test on a 3D-printed strip, where the burning occurred from the left side, the burned length ($L_{B}$) was measured to the lower left corner of the strip.

Indeed, the lower the linear burning rate (*v*), the more thermally stable the composite is in terms of flammability, indicating that it is more difficult to burn. It is worth noting that we conducted this test in a fume hood, where there is airflow from the outside to the inside of the hood. As a result, the burned length ($L_{B}$) was not uniform across the width of the strip, as depicted in Figure 3. After conducting the flammability test on a 3D-printed strip, where the burning occurred from the left side, the burned length ($L_{B}$) was measured to the lower left corner of the strip. To determine the burned length ($L_{B}$), we used the location of the fastest burned location along the strip.

#### 3.7.2. Thermogravimetric Analysis (Decomposition)

To investigate the thermal decomposition behavior of the composites and their complete degradation into gases upon heating, we employed a thermogravimetric analysis (TGA, NETZSCH STA449 F3 Jupiter, Selb, Germany). For this purpose, 50 mg of a pellet was placed in an open alumina crucible after pretreatment in the vacuum oven (OV11) at room temperature for over one week. TGA scans were conducted from 20 to 500 °C using a heating rate of 10 °C/min under a flowing N_2_ environment (0.02 L/min). The mass change of the samples during the TGA runs was recorded.

To assess the thermal stability, two variables were calculated. The residual mass percent (${Mass}_{R}$) was obtained by:

(2) $${Mass}_{R}\;\left(T\right)\;\left(\%\right)=\frac{M(T)}{M_{20{}^{\circ}C}}\times 100\%$$

To ensure a fair comparison among samples that have different ultimate residual masses at 500 °C, the normalized residual mass percent (${Mass}_{R,N}$) was calculated by:

(3) $${Mass}_{R,N}\left(T\right)\;(\%)=\frac{M(T)-M_{500{}^{\circ}C}}{M_{20{}^{\circ}C}-M_{500{}^{\circ}C}}\times 100\%$$

where M(T) is the sample mass at a certain temperature (*T*), $M_{20{}^{\circ}C}$ is the initial sample mass at 20 °C, and $M_{500{}^{\circ}C}\;$ is the sample mass at 500 °C. ${Mass}_{R,N}\left(T\right)=100\%\;at\;20\;{}^{\circ}C$, and ${Mass}_{R,N}\left(T\right)=0\%\;at\;500\;{}^{\circ}C$. The decrease in mass observed during the TGA runs can be attributed to the evaporation of gases resulting from the complete decomposition of the composite samples, particularly the PLA matrix. The residual mass represents the mass of the compounded NZ jade. Equation (3) is described as a means of fair comparison among samples with different ash content at 500 °C.

### 3.8. Time Sweep in Rotational Rheometry (Partial Degradation)

To characterize the rheological properties of the compression-molded composites, time sweep tests were performed using a rotational rheometer (SR5000, Rheometric Scientific, Piscataway, NJ, USA). Prior to the rheological tests, the compression-molded composite disks were stored in the vacuum oven (OV11) at room temperature for over a week to ensure consistent material properties. To minimize the effect of inertia caused by the upper plate, an inertia correction was applied before each test run without any sample disk.

During the time sweep test, a 1 mm-thick disk was placed between two parallel disks with a diameter of 25 mm. The time sweep test was conducted at a frequency (ω) of 10 rad/s and a strain (γ) of 0.1% and at a temperature of 215 °C in a N_2_ environment. This temperature was chosen to study the thermal degradation at the temperature at which the 3D printing process occurred. The duration of the test was 1 h.

During the time sweep test, we determined the storage modulus, $G^{\prime }(t)$, loss modulus, $G^{\prime \prime }(t)$, and eventually, the magnitude of the complex viscosity, $\left|\eta^{*}(t)\right|$ at different elapsed time (t) points. By monitoring the reduction in $\left|\eta^{*}(t)\right|$, we can infer the reduction in molecular weight due to thermal degradation.

To ensure a fair comparison among composites with different amounts of jade and their corresponding viscosities, a normalized magnitude of the complex viscosity is defined as follows:

(4) $$\eta_{n}^{*}(t)\equiv \frac{\left|\eta^{*}\left(t\right)\right|-\left|\eta^{*}\left(t_{final}\right)\right|}{\left|\eta^{*}\left(0\right)\right|-\left|\eta^{*}\left(t_{final}\right)\right|}$$

where $\eta_{n}^{*}=1$ in the beginning (t=0), $\eta_{n}^{*}=0$ at the end (${t=t}_{final}$) of the time sweep test, and t=3600 s for this study. This normalization allows us to assess the relative changes in viscosity independent of the specific amount of jade in each composite. The data of the normalized magnitude of the complex viscosity were fitted to the following model to quantify the speed of the thermal degradation, i.e., molecular weight decrease with time:

(5) $$\eta_{n}^{*}\left(t\right)=\frac{t_{1/2}\left(t_{final}-t\right)}{t_{final}\left(t_{1/2}+t\right)-2t_{1/2}t}$$

where $t_{1/2}$ is a fitting constant with physical meaning of the half time when the normalized magnitude of the complex viscosity reaches its initial value. This model shows $\eta_{n}^{*}(0)=1$, $\eta_{n}^{*}(t_{1/2})=0.5\eta_{n}^{*}\left(0\right)$, and $\eta_{n}^{*}\left(t_{final}\right)=0$. To compare the amount of the degradation quantitatively, the normalized total decrease in the magnitude of the complex viscosity ($\eta_{dec,n}^{*}$) is also defined:

(6) $$\eta_{dec,n}^{*}\equiv \frac{\left|\eta^{*}\left(0\right)\right|-\left|\eta^{*}\left(t_{final}\right)\right|}{\left|\eta^{*}\left(0\right)\right|}$$

Additionally, assuming the Cox-Merz rule [49,50], the viscosity as a function of shear rate $\left(\dot{\gamma }\right)$:

(7) $$\eta \left(\dot{\gamma }\right)=\left|\eta^{*}\left(\omega \right)\right|\;where\;\dot{\gamma }=\omega$$

can be inferred using $\left|\eta^{*}\left(\omega \right)\right|$ so that the molecular weight change with time based on Berry-Fox equation [51] is:

(8) $$\eta ={KM}_{w}^{a}$$

where $M_{w}$ is the weight-average molecular weight, $K=2.3\times 10^{-15}\;Pas$, and a=3.7 [52], can be inferred.

### 3.9. Mechanical Characterization

Initially, rheological frequency sweep tests were considered to obtain a flow curve by assuming the Cox-Merz rule. However, subsequent rheological time sweep tests revealed a rapid decrease in the magnitude of the complex viscosity for each composite over time. This indicates a significant change in viscosity during the course of the measurements, rendering the pursuit of flow curves impractical in this study. Two different types of tests were performed to investigate the mechanical strengths of the composites prepared via 3D printing and compression molding.

#### 3.9.1. Tensile Properties

Tensile tests [53] were conducted to determine the Young’s modulus ($E_{T}$), tensile strength ($\sigma_{TS}$), ultimate strain, and toughness of 3D-printed strips and compression-molded strips. The tests were performed in accordance with the ASTM D638-22 Standard Test Method for Tensile Properties of Plastics [54], using a universal test machine (UTM, model 5965 by Instron, Norwood, MA, USA). Before conducting the tensile tests, the strips were placed in the vacuum oven (OV11) at room temperature for over a week to ensure consistent material properties. Each strip was securely gripped vertically on both ends and subjected to tension until fracture occurred. The tests were conducted at a crosshead speed of 50 mm/s and a temperature of 20 °C. The tensile stress ($\sigma_{T}$) was determined using the formula:

(9) $$\sigma_{T}=\frac{F_{T}}{wd}$$

where $F_{T}$ is the tensile force, w is the width of the strip, and d is the thickness of the strip. The tensile strain ($\varepsilon_{T}$) was obtained by:

(10) $$\varepsilon_{T}=\frac{\Delta L}{L_{0}}$$

where ΔL is the tensile displacement, $L_{0}$ is the initial length of the strip, and the ultimate tensile strain is the maximum strain when the sample breaks. The tensile strength ($\sigma_{TS}$) is the maximum tensile stress:

(11) $$\sigma_{TS}=\frac{F_{T,max}}{wd}$$

where $F_{T,\;max}$ is the maximum tensile force applied. The Young’s (elastic) modulus ($E_{T}$) was determined as the slope of the $\sigma_{T}$ versus $\varepsilon_{T}$ plot within its linear regime:

(12) $$E_{T}=\frac{{\Delta \sigma }_{T}}{\Delta \varepsilon_{T}}$$

The toughness of the material was determined by calculating the total area under the curve of $\sigma_{T}$ versus $\varepsilon_{T}$. These measurements allowed for the characterization of important mechanical properties of the composite strips, providing insights into their strength and stiffness under tensile loading and comparison between 3D-printed and compression-molded samples.

#### 3.9.2. Three-Point Bending

To determine the flexural strength and flexural modulus of the 3D-printed strips and compression-molded strips, three-point bending tests were conducted following the ASTM D790-17 Standard Test Methods for Flexural Properties of Unreinforced and Reinforced Plastics and Electrical Insulating Materials [55], using the UTM. Prior to the tests, the strips underwent vacuum oven (OV11) treatment at room temperature for over a week to ensure consistent material properties. During the three-point bend tests, each strip was placed horizontally on two fixed supports with a span of 40 mm between them. The strip was loaded at its center in compression at a speed of 3 mm/min and at a temperature of 20 °C until fracture occurred. The flexural stress ($\sigma_{F}$) was determined using the formula:

(13) $$\sigma_{F}=\frac{3F_{F}S}{2wd^{2}}$$

where $F_{F}$ is the downward force and S is the distance between the two supports (i.e., the supports span). The flexural strain ($\varepsilon_{F}$) was obtained by:

(14) $$\varepsilon_{F}=\frac{6\delta d}{S^{2}}$$

where δ is the deflection at the center of the strip. The flexural strength ($\sigma_{FS}$) was attained by:

(15) $$\sigma_{FS}=\frac{3F_{F,max}S}{2wd^{2}}$$

where $F_{F,\;max}$ is the maximum downward force applied. The flexural (bending) modulus ($E_{F}$) was determined as the slope of the $\sigma_{F}$ versus $\varepsilon_{F}$ plot within its linear regime using:

(16) $$E_{F}=\frac{{d\sigma }_{F}}{d\varepsilon_{F}}$$

### 3.10. Statistical Analysis

The statistical analysis was conducted using one-sided Student *t*-tests in Microsoft Excel 2019. Data sets with *p*-values below 0.05 were deemed statistically significant. In the plots, significance levels were denoted as * for *p* < 0.05, ** for *p* < 0.01, and *** for *p* < 0.001. The number of repetitions for each sample is specified in the respective sections.

## 4. Results and Discussion

### 4.1. NZ Jade

**Figure 4:**
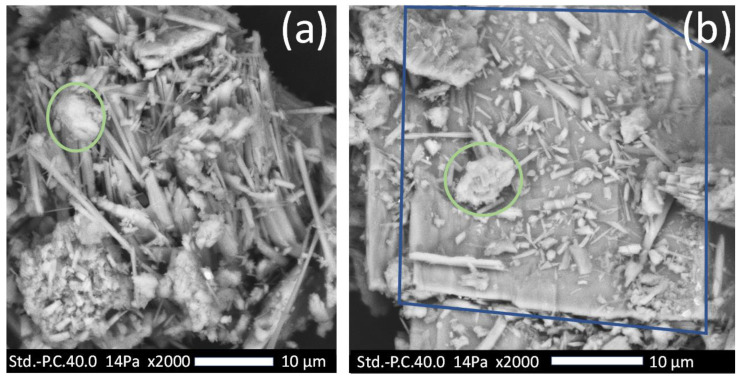
SEM images of NZ jade particles: (**a**) Chunk (green circle) and needle structures; (**b**) chunk (green circle), long needle, short needles, and plate (blue box) structures.

Figure 4 displays SEM images of NZ jade particles, showcasing diverse structures, including chunks (indicated by green circles), needles, and plate-like structures (enclosed in the blue box). Some of these structures appear fused together or in proximity. The composition of the plate-like structures, whether they consist of needles, remains unclear. However, it seems that the shorter needles may have originated from the longer ones through the grinding process. The grinding process of NZ jade rock results in ground pieces of varying sizes, highlighting the importance of screening for the desired sizes to ensure consistency. In Table 4, it is evident that the supplier provides a majority of particles measuring 38 µm or smaller. It is imperative to wear personal protective equipment, such as masks and goggles, when grinding or handling these particles, particularly due to the slender width (<1 µm) of the needles. 

**Figure 5:**
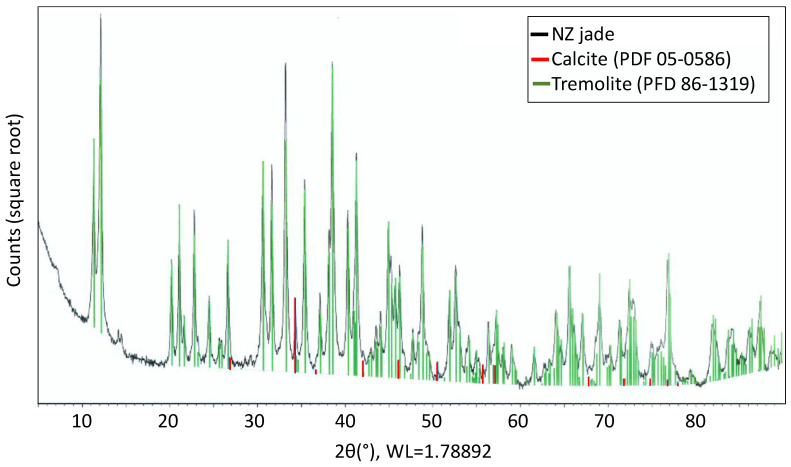
XRD spectra of NZ jade, calcite, and tremolite. The legend for the curve gives the PDF number and the mineral names that best match the families of peaks observed.

Considering the expected elements present in NZ jade, the peaks were compared with the particle diffraction files (PDFs) [56] available in the International Centre for Diffraction Data (ICDD) for well-characterized minerals. Figure 5 displays the XRD spectra of NZ jade along with reference peaks of calcite (CaCO_3_, PDF 05-0586) and tremolite [(${Ca}_{1.97}{Na}_{0.016}{Mg}_{5}{Fe}_{0.014}^{2+}){Si}_{8}O_{22}{\left(OH\right)}_{2}$, PDF 86-1319] based on their respective PDFs. The small peaks observed at approximately 14° of 2θ are not fully explained since it is challenging to reference only two peaks (as many inorganic samples have tens to hundreds of peaks). However, these peaks may partially result from the presence of a superstructure in the tremolite phase of NZ jade. Additionally, there is a suggestion of a trace amount of 14 angstrom clay present (at 7° of 2θ), indicated by the expected appearance of the 002 clay peak at approximately 14° of 2θ. A small amount of calcite is also detected, which accounts for the slight white tone, rather than pure green, observed in NZ jade. The principal constituent of NZ jade is tremolite, as most of the peaks of these two materials overlap. Table 5 and Table 6 present the compositions of elements in NZ jade measured by EDAX, as well as the compositions of oxide compounds measured by XRF. The molar percentage of each element and compound is consistent with the stoichiometry of elements and compounds in tremolite [57]. Assuming that the content of calcite is negligible, the elemental ratio Ca/Mg/Fe/Si = 2:4.5:0.6:8, indicating that both the iron and magnesium contents are relatively high, resembling tremolite. It is known that tremolite exhibits a green coloration with increased iron content and a white coloration with higher magnesium content, thus explaining the green-white color observed in the NZ jade used in this study. In particular, a green variety of tremolite is referred to as nephrite [58].

The greenish color of NZ jade primarily stems from its microscopic needle-like structure, consisting of tightly interwoven fibers of actinolite, an amphibole mineral. The presence of iron within the actinolite fibers contributes to the green coloration observed in NZ jade. These iron impurities impart the stone with its characteristic green hue. However, it is important to note that not all New Zealand jade exhibits a uniform green color, as XRD data (Figure 5) and structures (Figure 4) indicate the presence of other minerals. NZ jade showcases various variations and shades of green, ranging from pale green to deep green (Figure 1), and may also display colors such as white and brown.

### 4.2. Compounded Strips

**Figure 6:**
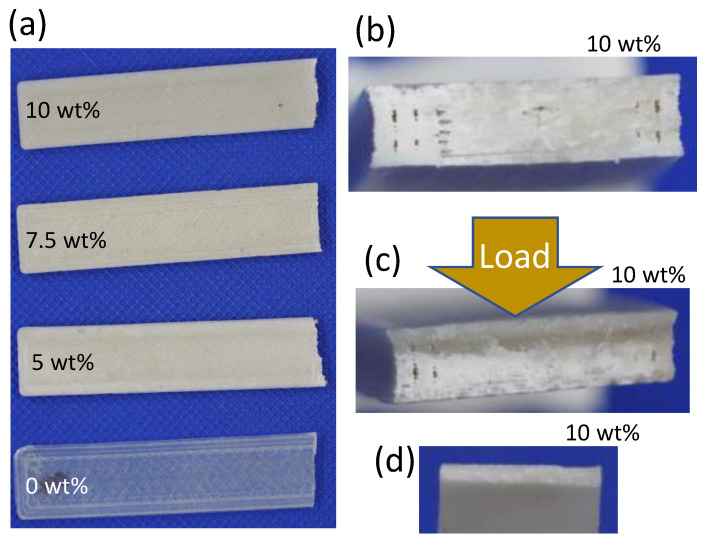
Appearance of: (**a**) 3D-printed strips after tensile tests; (**b**) failure cross-section of 3D-printed strip after a tensile test; (**c**) failure cross-section of 3D-printed strip after a 3-point bending test; (**d**) failure cross-section of compression-molded strip after a tensile test. (The darker face is the side of the strip.) Images are not to scale.

Table 7 presents the density values for both compression-molded and 3D-printed strips. The density increases with the amount of compounded jade particles. Specifically, the density of compression-molded strips is 2–13% higher compared to that of 3D-printed strips. These findings align with expectations, as the applied pressure in compression molding minimizes the gap between polymer melts, whereas no such pressure is present during 3D printing even though the strips were 3D-printed with the 100% infill condition. It is worth noting that this difference in density will have an impact on the mechanical properties, which will be discussed in detail later. In Figure 6, the appearance of both compression-molded and 3D-printed strips is illustrated. The color gradually darkens with an increasing jade content, but no significant differences are observed beyond that.

### 4.3. Thermal Stability of Composites

#### 4.3.1. Flammability

**Figure 7:**
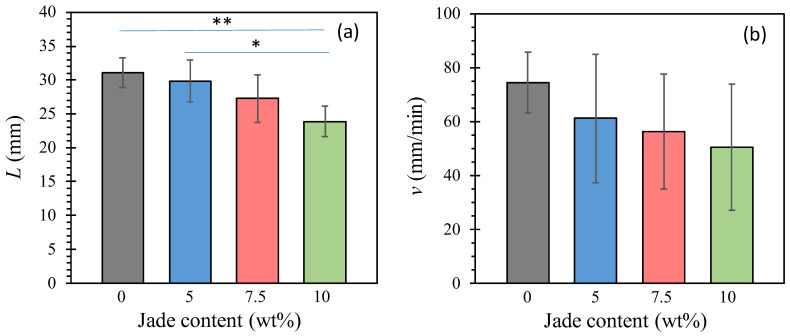
Flammability of 3D-printed composites: (**a**) Burned length; (**b**) linear burning rate. *, *p* < 0.05 and **, *p* < 0.01.

Poly(lactic acid) (PLA) is known to be a flammable plastic; therefore, it is crucial to identify additives that can enhance flame retardation, i.e., reduce the flammability of the composites [59]. In our study, we investigated the impact of NZ jade content on the flammability of 3D-printed composites in flammability tests. During these tests, the flames ceased before reaching the 100 mm mark, allowing us to obtain $L_{B}\;and\;V$ [Equation (1)] from the point where flame propagation stopped. Figure 7 illustrates the flammability behavior of the composites. In Figure 7a, the 10 wt% composite exhibited a 25% reduction in burned length compared to the 0 wt% composite. While only the 10 wt% sample showed a statistically significant decrease compared to the 0 and 5 wt% composites, there is a gradual decrease observed with increasing NZ jade content. This result is expected since a higher NZ jade content results in less material available to burn within the given sample dimensions.

On the other hand, there are various options available to enhance the flame retardancy of PLA [59] such as the use of talc [40], and NZ jade can be considered as one of these options. On the other hand, an increase in NZ jade content did not lead to a statistically significant decrease in the linear burning rate [Figure 7b], primarily due to the relatively high standard deviation in the flame time measurements. However, a gradual decrease can be observed, and the 10 wt% composite exhibited a 30% reduction in burning rate compared to neat PLA. It is common practice to incorporate flame or fire retardants into the polymer matrix to achieve flame retardation [60,61,62]. There are a few different mechanisms in flame retardation including endothermic degradation [63] (where some compounds break down endothermically when subjected to high temperatures, cooling the substrate and the flame, delaying ignition), thermal shielding [64] (which is a way to stop the spread of flame over the material by creating a thermal insulation layer between the burning and unburned parts), dilution [65] (which is the use of inert gases, most commonly carbon dioxide and water vapor, released by flame retardants can dilute the fuel in the flame, reducing its heat output and slowing its growth), and gas phase radical quenching [66] (which is the use of halogenated flame retardants releasing hydrogen chloride or hydrogen bromide upon heating, and reacting with high-energy radicals in the flame, forming stable molecules that do not participate in the chain reaction). Considering the inert nature of NZ jade, thermal shielding is the most likely mechanism at play.

#### 4.3.2. Thermal Decomposition

**Figure 8:**
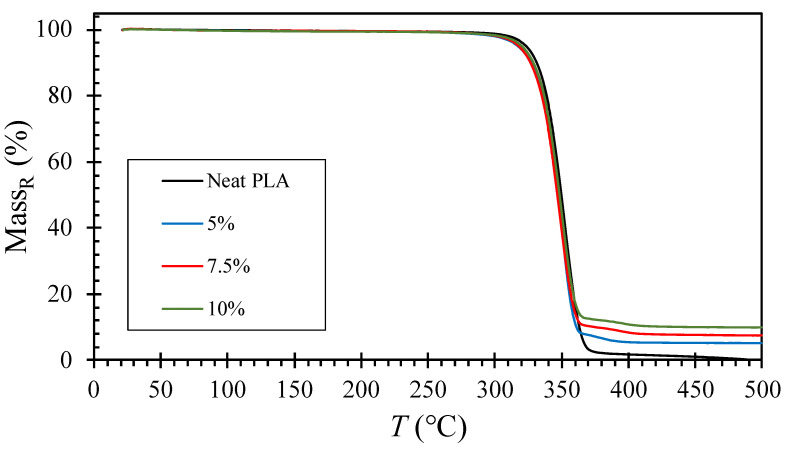
Residual mass percent from TGA runs from 20 to 500 °C at 10 °C/min. The percentages are the content of NZ jade in wt%.

**Figure 9:**
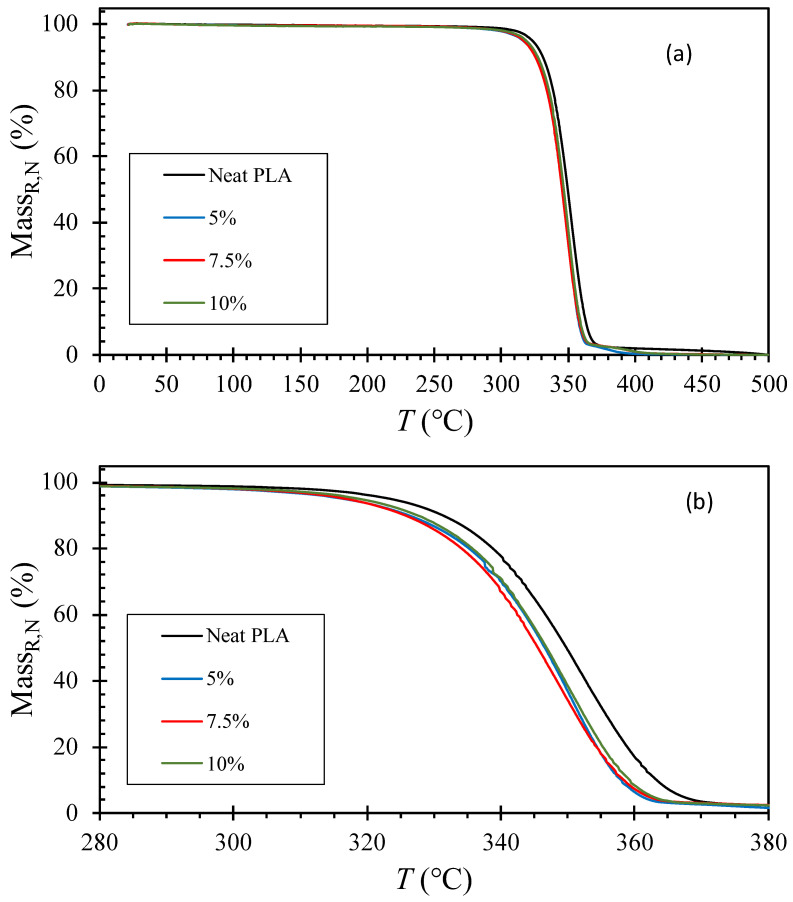
Normalized residual mass percent from TGA runs from 20 to 500 °C at 10 °C/min: (**a**) Whole range; (**b**) zoomed in range where the apparent drops are observed. The percentages are the content of NZ jade in wt%.

Figure 8 shows the residual mass percent [Equation (2)] from TGA runs from 20 to 500 °C at a heating rate of 10 °C/min for each sample. The neat PLA exhibited 0% residual mass at 500 °C, indicating the absence of ash content, while the composite samples showed a residual mass at 500 °C, which correlated with the mass content of the compounded NZ jade. To compare the onset temperature of evaporation, the normalized residual mass percent [Equation (3)] is a more appropriate measure, as depicted in Figure 9. All plots reached 0% at 500 °C [Figure 9a], and the zoomed in plots [Figure 9b] allow for a comparison of the onset and completion of evaporation. It is evident that the mass of neat PLA decreases significantly at higher temperatures compared to NZ jade-compounded PLAs. The differences among the jade-compounded PLAs are insignificant, with most of the evaporation completing around 365 °C, while neat PLA completes at approximately 370 °C. Thus, Figure 9b demonstrates that the thermal decomposition of jade-compounded PLA initiates and concludes at a lower temperature, approximately 5 °C lower than neat PLA. Compounding NZ jade leads to a reduction in thermal stability, regardless of the composition. However, it is worth noting that Yu et al. [64] demonstrated that compounding talc with PLA enhanced thermal stability by increasing the decomposition temperature in TGA by 1 or 2 °C. Therefore, our results show the opposite trend. It is possible to hypothesize that compounded NZ jade particles provided reactive sites for degradation. Nonetheless, the exact cause of this trend is unknown. However, the trends observed in thermal decomposition and flammability suggest the positive potential for reduced flammability and accelerated biodegradation by incorporating jade. Further discussion on these topics will be presented in the next section.

#### 4.3.3. Thermal Molecular Weight Decrease

One of the challenges with biodegradable plastics is their slow biodegradation, as mentioned earlier. Therefore, it is valuable to investigate whether the addition of NZ jade to the resin can accelerate biodegradation. However, confirming biodegradation and its kinetics typically requires several months, making it necessary to find a method to predict or compare degradation kinetics using a relatively simple protocol. While TGA data provide information on thermal stability and complete decomposition of the polymer matrix into gases, they do not indicate how the molecular weight changes over time. Conducting gel permeation chromatography (GPC) for samples obtained at each minute at high temperatures could be an option, but this would not capture the actual properties at the sampling time since changes can still occur during sampling. Additionally, running GPC for so many samples would be impractical. Hence, GPC is not a suitable method for tracking real-time molecular weight changes at high temperatures.

**Figure 10:**
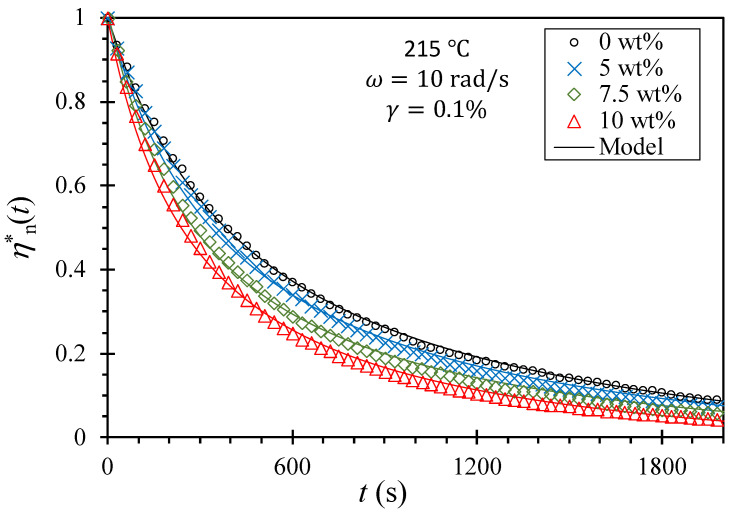
The normalized magnitude of the complex viscosity [Equation (4)] with time, and solid curves are the model predictions [Equation (5)]. To avoid confusion, plots only up to 2000 s are shown.

On the other hand, we can infer molecular weight decreases from rheological time sweep data [Equation (8)] at relatively high temperatures, which provides an alternative perspective on thermal stability that is relevant to the potential for biodegradation kinetics. We employed rheometry to compare the kinetics of molecular weight decrease due to thermal degradation for each composite. Specifically, dynamic time sweep tests were conducted at a frequency (ω) of 10 rad/s, strain (γ) of 0.1%, and at a temperature of 215 °C. Those tests proclaimed all the rheological variables, $G^{\prime }(t)$, $G^{\prime \prime }(t)$, and $\left|\eta^{*}(t)\right|$, decrease over time, and the decrease reaches a saturation point before 1 h of elapsed (annealing) time. Since each composite exhibits different initial and final values of these variables in the time sweep, the normalized magnitude of the complex viscosity, $\eta_{n}^{*}\left(t\right)$ [Equation (4)] was calculated and plotted in Figure 10. Then, these values were fitted to the model (Equation (5), solid curves in Figure 10). It is important to note that $\eta_{n}^{*}\left(t\right)$ starts to decrease from the beginning of the time sweep even though all rheological measurements were performed in a nitrogen environment using a vacuum-dried disk, and this suggests that only a small quantity of stabilizers was added to the resin.

**Figure 11:**
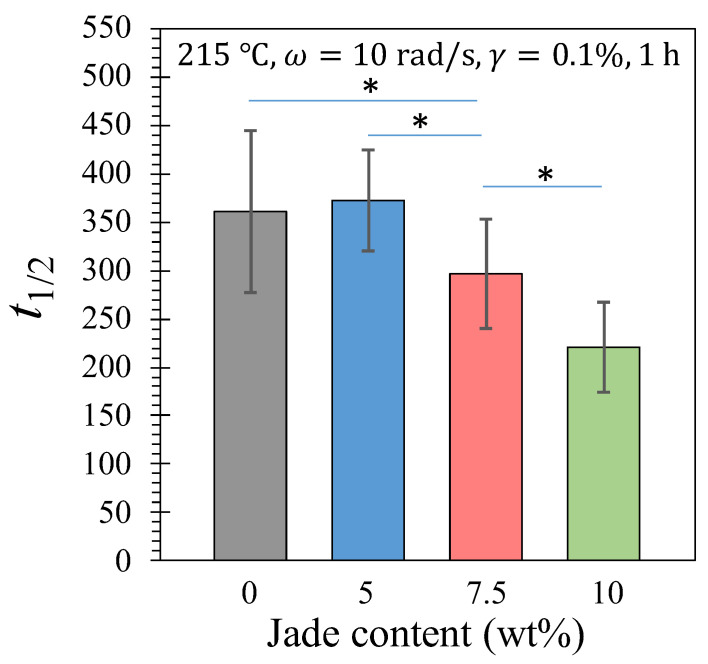
The half-time for $\eta_{n}^{*}\left(t\right)$ to drop to half the initial value at 215 °C. Three repeats for each composite were used for the average, and error bars is the standard deviation. *, *p* < 0.05.

Even though Equation (5) only requires one fitting parameter, $t_{1/2}$, the model prediction aligns reasonably well with the experimental data. To quantitatively compare the degradation kinetics, $t_{1/2}$ for $\eta_{n}^{*}\left(t\right)$ to decrease to half of its initial value is presented in Figure 11. While there is no significant difference between the 0 and 5 wt% composites, a discernible trend of decreasing half-time with increasing NZ jade content can be observed. Therefore, it is reasonable to expect that the thermal degradation can be further accelerated by incorporating higher amounts of NZ jade in the composite.

**Figure 12:**
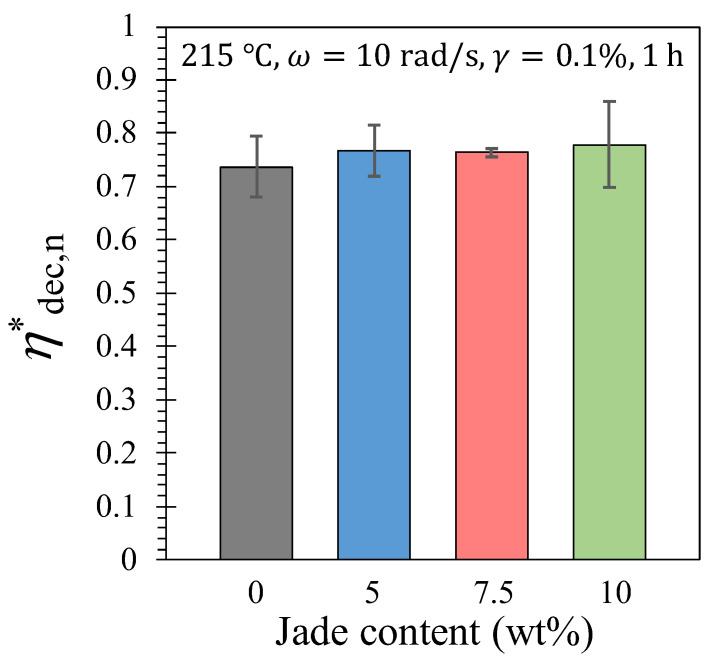
The normalized total decreases in the magnitude of the complex viscosity after 1 h at 215 °C. Three repeats for each composite were used for the average and error bars, which is standard deviation.

Figure 12 illustrates the normalized total decrease in the magnitude of the complex viscosity, $\eta_{dec,n}^{*}$ [Equation (6)]. Although there is a slight trend of increasing values with higher NZ jade content, the difference is not statistically significant. This indicates that compounded NZ jade accelerates thermal degradation without significantly impacting the overall extent of degradation. The magnitude of the complex viscosity decreases by 75% from its initial value for all the composites. To estimate the change in molecular weight, we can utilize the following equation derived from rearranging Equation (8):

(17) $$\frac{M_{w,f}}{M_{w,i\;}}=10^{(1/3.7)\log\left(\eta_{f}/\eta_{i}\right)}=0.69$$

where $M_{w,f}$ is the molecular weight at the end of time sweep, $M_{w,i}$ is the molecular weight at the beginning of time sweep, $\eta_{f}$ is the viscosity at the end of time sweep, $\eta_{i}$ is the viscosity at the beginning of time sweep, and $\eta_{f}/\eta_{i}=0.25$ (as observed in Figure 10). According to Equation (17), the molecular weight experiences a decrease of approximately 30% due to the addition of NZ jade after 1 h at 215 °C in a nitrogen environment.

It is important to note that it is premature to conclude that adding jade particles will directly accelerate the biodegradation of the biodegradable plastic matrix as the mechanisms of thermal degradation and biodegradation are distinct. However, the significant reduction in molecular weight due to thermal degradation observed is encouraging for potential accelerated biodegradation. It is known that biodegradation can occur more rapidly on shorter polymeric chains of PLA [67]. Therefore, the composites containing NZ jade, which undergo faster thermal degradation and generate shorter polymer molecules, may experience accelerated biodegradation compared to neat PLA. Future research should focus on investigating the effect of NZ jade compounding on the biodegradation of these composites.

The choice of 215 °C for the time sweep tests aligns with the temperature used during the 3D printing process, indicating that similar degradation would occur during printing at that temperature. Although the printing time is relatively short, there is a significant decrease in viscosity at the beginning of the time sweep (Figure 10) suggesting that the resulting 3D-printed part may have a lower molecular weight compared to the filament and reduced mechanical strength. To examine this effect, tensile tests were conducted on both 3D-printed and compression-molded strips, as described in the subsequent section.

### 4.4. Mechanical Properties

#### 4.4.1. Tensile Properties

The tensile tests conducted provide valuable insights into the mechanical properties of the composites, including stiffness, strength, ductility, and toughness, which are crucial for assessing their performance under linear stretching conditions and suitability for specific applications. These tests aimed to investigate how the addition of NZ jade and the preparation processes (3D printing versus compression molding) influence the mechanical performance of the composites under bending load.

**Figure 13:**
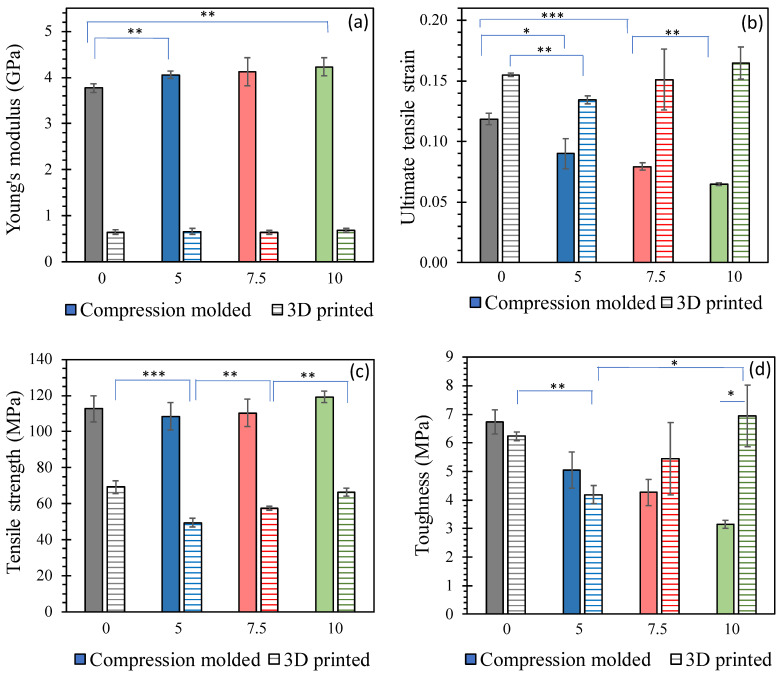
Tensile test results. Solid bar graphs are for compression-molded composites, while striped bar graphs are for 3D-printed composites. Three repeats for each composite were used for the average and error bars, which is standard deviation. The numbers on x-axis are the content of NZ jade in wt%: (**a**) Young’s modulus; (**b**) ultimate tensile strain; (**c**) tensile strength; (**d**) toughness. *, *p* < 0.05; **, *p* < 0.01; and ***, *p* < 0.001.

Figure 13a demonstrates that the Young’s modulus, which indicates the stiffness of the material, increases with the jade content for compression-molded strips. However, there is no significant difference observed among the 3D-printed composites. Notably, the 3D-printed composites exhibit a Young’s modulus that is six times lower than that of the compression-molded ones, suggesting that 3D printing results in a softer and more ductile product compared to compression molding. This can be attributed to two reasons. Firstly, the failure cross-section analysis depicted in Figure 6b,d reveals that 3D-printed strips exhibit dark cavities, which likely occurred during the 3D printing process. These defects contribute to reduced density (Table 7) and, consequently, soften the composites. Secondly, the 3D printing process is susceptible to inducing thermal oxidative degradation of the polymer [68] leading to a decrease in molecular weight and further softening of the material. This softening effect dominates, resulting in no significant change in Young’s modulus with jade content for the 3D-printed composites. Figure 13b presents the ultimate tensile strain, which indicates the maximum stretchability of the material. The 3D-printed strips exhibit higher ultimate tensile strain compared to the compression-molded strips, and this difference increases with the jade content. This difference should be due to the softened structure. The ultimate tensile strain of the compression-molded strips decreases with increasing jade content, mainly because of the jade particles, which make the strips less stretchable.

Tensile strength, as shown in Figure 13c, follows a similar trend as Young’s modulus [Figure 13a]. The compression-molded strips exhibit approximately twice the tensile strength of the 3D-printed strips. The 3D-printing process not only results in a softer structure but also weakens the material, leading to lower tensile strength. The tensile strength gradually increases with jade content after a slight drop at 5 wt% jade. Although this increase is not statistically significant for the compression-molded composites, it is significant for the 3D-printed composites. Figure 13d illustrates the toughness of the composites, which represents their ability to absorb energy before fracture. The toughness of the compression-molded strips continuously decreases with jade content, indicating increased brittleness. In contrast, the 3D-printed composite exhibits similar toughness compared to the compression-molded composites, except for the 10 wt% jade sample, where toughness increases with jade content after a drop at 5 wt%. Thus, 3D printing may be a better process for applications requiring a higher toughness. Overall, the tensile test results highlight the different mechanical behaviors of the composites depending on the presence of NZ jade and the preparation process. 3D printing leads to softer and more ductile composites, while compression molding results in stiffer but more brittle composites. The addition of NZ jade affects the mechanical properties differently in the two processes, with varying levels of significance.

A study by Yu et al. [69] demonstrated that compounding talc with PLA improved various mechanical properties, including tensile strength, toughness, Young’s modulus, flexural strength, and flexural modulus, up to a certain talc content. They also observed an increase in ultimate strain up to a specific talc content. It is reasonable to expect similar effects when compounding NZ jade with PLA due to similar mechanisms as in the PLA/talc system. The authors of that study attributed the improved mechanical properties to enhanced crystallinity and accelerated crystallization kinetics. Similarly, De Santis and Pantani [70] investigated the effects of compounded talc in PLA and found that it reduced both the crystallization time and temperature. While the current study did not focus on studying crystallization kinetics, it is plausible to assume that similar mechanisms could be at play in the case of PLA/jade composites. The addition of jade particles provides nucleation sites for crystallization, which can lead to enhanced mechanical properties. Therefore, based on the existing literature, it is reasonable to expect that compounding NZ jade with PLA can result in improved mechanical properties, similar to the PLA/talc system, through enhanced crystallinity and accelerated crystallization kinetics. However, further studies would be required to investigate the specific effects of jade particles on the crystallization behavior and mechanical properties of PLA.

#### 4.4.2. Three-Point Bending Properties

The three-point bending tests were conducted to evaluate the mechanical properties of the composites under bending conditions. These tests provide important information about the stiffness, strength, and ductility of the materials, which is essential for assessing their performance in bending applications. The effects of compounded NZ jade and the preparation processes (compression molding versus 3D printing) on the mechanical performance were investigated.

**Figure 14:**
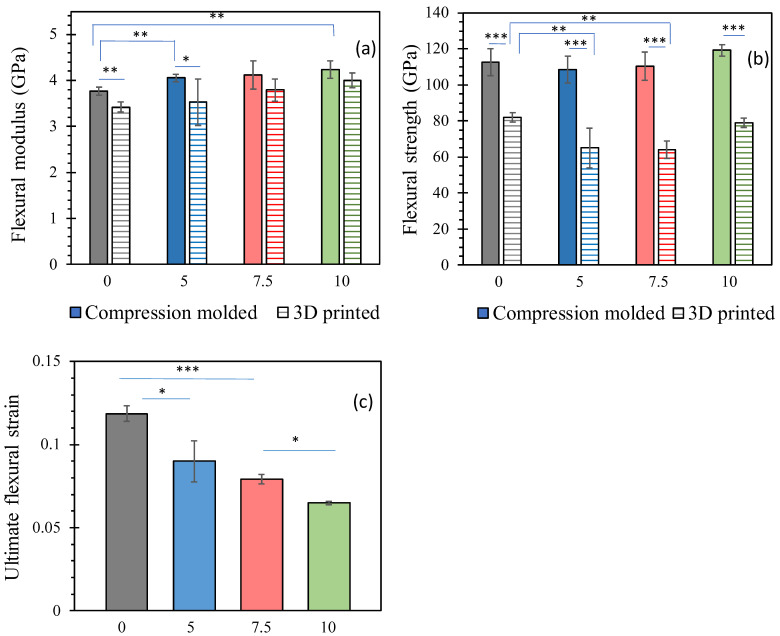
Three-point bending test results. Solid bar graphs are for compression-molded composites, while striped bar graphs are for 3D-printed composites. Three repeats for each composite were used for the average and error bars, which is standard deviation. The numbers on x-axis are the content of NZ jade in wt%: (**a**) Flexural modulus; (**b**) flexural strength; (**c**) ultimate flexural strain. *, *p* < 0.05; **, *p* < 0.01; and ***, *p* < 0.001.

Figure 6c shows the failure cross-section of a 3D-printed strip after a three-point bending test. Similar to the tensile tests, the presence of dark cavities can be observed, indicating the occurrence of defects during the 3D printing process. Figure 14 presents the results of the bending tests. The flexural modulus, shown in Figure 14a, generally increases with the content of NZ jade for both compression-molded and 3D-printed strips. The addition of stiff jade particles enhances the overall stiffness of the composites when subjected to bending. However, it is worth noting that 3D-printed strips exhibit slightly (5–10%) lower flexural modulus compared to compression-molded ones, but this difference is much smaller than the disparity observed in Young’s modulus from the tensile tests in Figure 13a, indicating that the effects of the two preparation processes manifest differently under different types of deformation. The flexural strength [Figure 14b] shows a significant difference between the two preparation processes. 3D-printed strips exhibit 30–40% lower flexural strength compared to compression-molded ones. The harsher conditions during filament formation and printing in an aerobic environment likely contribute to lower molecular weight, density, and strength in 3D-printed composites. Additionally, the flexural strength of 3D-printed strips is more affected by the addition of NZ jade, while the impact on compression-molded strips is relatively minimal. This could be attributed to the reduced physical bonding [71] between PLA and jade particles in 3D-printed composites due to thermal degradation during the printing process. Figure 14c shows that the ultimate flexural strain of compression-molded strips decreases with increasing jade content, similarly to the trend observed in ultimate tensile strain [Figure 13b]. A higher concentration of jade particles leads to decreased ductility in the composites. This can be attributed to the fact that the solid jade particles do not undergo stretching, and any weak interfaces between the particles and the PLA matrix can act as sites for rupture.

In summary, the three-point bending tests reveal that the addition of NZ jade affects the flexural modulus and strength of the composites, with the effects being more pronounced in 3D-printed strips. The presence of defects and reduced molecular weight in 3D-printed composites contribute to lower mechanical strength. The ultimate flexural strain decreases with increased jade content, indicating reduced ductility in the composites. These findings highlight the importance of considering the processing conditions and material properties when utilizing 3D printing for biodegradable plastics, as they may be more susceptible to thermal degradation and exhibit altered mechanical performance compared to compression-molded counterparts.

## 5. Conclusions

We investigated the effects of compounding PLA (polylactic acid) with NZ jade particles, with a particle size of 38 µm or smaller, at various weight percentages (0, 5, 7.5, and 10 wt%) using a twin-screw extruder. Two types of samples were fabricated: 3D-printed and compression-molded. Our findings demonstrate that the incorporation of jade particles has significant impacts on the density, thermal stability, and mechanical properties of the composites.

Regarding density, the addition of jade particles resulted in a noticeable increase in the overall density of the composites. This increase can be attributed to the higher density of the jade particles compared to PLA.

Furthermore, the compounded jade particles influenced the thermal stability of the composites. An important aspect we examined was the flame retardation (flammability) of the composites. Remarkably, the addition of jade particles led to an increase in flame retardation (i.e., a reduction in flammability), indicating that these particles may possess flame-retardant properties. This finding has significant implications for the potential use of jade-based composites in applications where fire resistance is crucial.

Interestingly, the addition of jade accelerated the thermal degradation process in terms of molecular weight decrease and decomposition temperature reduction. Considering the biodegradability of the composites, our results suggest that the presence of jade particles could potentially accelerate the biodegradation process. However, it is important to note that further research is necessary to thoroughly investigate the biodegradation behavior of these composites and to determine the extent of the impact of jade particles on the overall biodegradability.

The effects of compounding jade particles on mechanical properties were found to be complex. Notably, we observed differences between the compression-molded and 3D-printed samples. The compression-molded composites exhibited higher Young’s and flexural moduli as well as greater tensile strength, compared to the 3D-printed composites. This suggests that the compression molding process produces stronger and stiffer composites compared to the 3D printing technique. The mechanical properties of the compression-molded composites were found to improve with increasing jade content. Specifically, higher jade content resulted in higher Young’s and flexural moduli, as well as increased tensile and flexural strengths. Therefore, it can be inferred that compression-molded products with higher jade content would exhibit enhanced strength and stiffness.

## Figures and Tables

**Table 1:** Composites formulation for extrusion compounding.

Code	PLA (wt%)	NZ Jade Particles (wt%)
0 wt%	100.0	0.0
5 wt%	95.0	5.0
7.5 wt%	92.5	7.5
10 wt%	90.0	10.0

**Table 2:** Conditions for extrusion compounding.

Temperatures (die to feed) (°C)	185–190
Screw speed (rpm)	180
Feed speed (rpm)	25–30 depending on the formulation
Pelletizer speed (m/min)	17.5
Pellets diameter (mm)	2.5
Running pressure (bar)	30–45 depending on the formulation

**Table 3:** Conditions for filament production.

Temperatures (die to feed) (°C)	150–190
Screw speed (rpm)	150
Feed speed (rpm)	20–30 depending on the formulation
Caterpillar speed (m/min)	25
Running pressure (bar)	50–70 depending on the formulation

**Table 4:** Particle size distribution after screening.

Screen Size (µm)	wt%
>212	0.12
106–212	0.30
75–106	1.09
38–75	2.46
0–38	96.04

**Table 5:** Average composition of elements on four NZ jade particles measured by EDAX. The standard deviation is below 1%.

Element	wt%	mol%
O	49.5	64.5
Si	24.8	18.4
Mg	12.3	10.6
Ca	8.7	4.5
Fe	3.8	1.4
Al	0.7	0.5

**Table 6:** Composition of compounds on NZ jade particles measured by XRF.

Compound	wt%	mol%
SiO_2_	60.0	57.6
Al_2_O_3_	0.2	0.10
Fe_2_O_3_	5.7	2.1
MnO	0.15	0.12
MgO	18.4	26.3
CaO	13.3	13.7
Na_2_O	0.04	0.04
P_2_O_5_	0.04	0.01

**Table 7:** Density of 3D-printed strips based on three repeats. The standard deviation is below 0.02 g/cc.

wt% of NZ Jade	Density (g/cc)		Density of Compression-Molded/Density of 3D-Printed
	Compression-Molded	3D-Printed	
0	1.31	1.28	1.02
5	1.41	1.29	1.09
7.5	1.47	1.30	1.13
10	1.50	1.33	1.13

## Data Availability

The data presented in this study are available on request from the corresponding author.
